# Impact of concomitant injuries on outcomes after traumatic brain injury

**DOI:** 10.1007/s00402-013-1710-0

**Published:** 2013-03-05

**Authors:** Johannes Leitgeb, Walter Mauritz, Alexandra Brazinova, Marek Majdan, Ingrid Wilbacher

**Affiliations:** 1 Department of Traumatology, Medical University of Vienna, Währinger Gürtel 18-20, 1090 Vienna, Austria; 2 Anesthesiology and Intensive Care Medicine, Trauma Hospital “Lorenz Boehler”, Vienna, Austria; 3 International Neurotrauma Research Organization (INRO), Vienna, Austria; 4 Department of Public Health, Faculty of Health and Social Services, Trnava University, Trnava, Slovak Republic

**Keywords:** Traumatic brain injury, Outcome, Concomitant injuries

## Abstract

**Background:**

Patients with traumatic brain injury (TBI) frequently have concomitant injuries; we aimed to investigate their impact on outcomes.

**Methods:**

Between February 2002 and April 2010, 17 Austrian centers prospectively enrolled 863 patients with moderate and severe TBI into observational studies. Data on accident, treatment, and outcomes were collected. Patients who survived until intensive care unit (ICU) admission and had survivable TBI were selected, and were assigned to “isolated TBI” or “TBI + injury” groups. Six-month outcomes were classified as “favorable” if Glasgow Outcome Scale (GOS) scores were five or four, and were classified as “unfavorable” if GOS scores were three or less. Univariate statistics (Fisher’s exact test, *t* test, χ^2^-test) and logistic regression were used to identify factors associated with hospital mortality and unfavorable outcome.

**Results:**

Of the 767 patients, 403 (52.5 %) had isolated TBI, 364 (47.5 %) had concomitant injuries. Patients with isolated TBI had higher mean age (53 vs. 44 years, *P* = 0.001); hospital mortality (30.0 vs. 27.2 %, *P* = 0.42) and rate of unfavorable outcome (50.4 vs. 41.8 %, *P* = 0.02) were higher, too. There were no significant mortality differences for factors like age groups, trauma mechanisms, neurologic status, CT findings, or treatment factors. Concomitant injuries were associated with higher mortality (33.3 vs. 12.5 %, *P* = 0.05) in patients with moderate TBI, and were significantly associated with more ventilation, ICU, and hospitals days. Logistic regression revealed that age, Glasgow Coma Scale score, pupillary reactivity, severity of TBI and CT score were the main factors that influenced outcomes.

**Conclusions:**

Concomitant injuries have a significant effect upon the mortality of patients with moderate TBI. They do not affect the mortality in patients with severe TBI.

**Level of evidence and study type:**

Evidence level 2; prospective, observational prognostic study.

## Introduction

A significant number of patients with traumatic brain injury (TBI) have concomitant injuries. These injuries may vary in severity, and their impact may vary accordingly. It has been reported that 40 % of the patients with severe TBI die from non-neurological causes, with higher incidence in patients with multiple injuries [1]. A German analysis found that mortality after head injury was 5 % higher in patients with severe concomitant injuries [2]. Another study reported that in patients with epidural hematoma (EDH) outcomes were not worsened by the presence extracranial injuries [3]. The goal of this study was to investigate the influence of concomitant injuries upon the outcomes of patients with moderate to severe TBI. Our hypothesis was that concomitant injuries would increase mortality after TBI.

## Patients and methods

Between 2001 and 2010, the International Neurotrauma Research Organization (INRO, founded 1999; based in Vienna, Austria) coordinated two projects that focused on Austrian patients with TBI. Databases developed by INRO were used to collect data for both projects. In the first project epidemiology and hospital treatment of patients with severe TBI as well as the effects of guideline-based treatment were analyzed [4]. This project started in March 2002; five centers enrolled 415 patients until June 2005. The second project focused on prehospital and early hospital management of patients with moderate and severe TBI. It started in March 2009; 16 centers enrolled 448 patients until April 2010. Both projects were done with approval of the local Ethical Committees.

### Centers

Seventeen Austrian centers participated in these projects, all were of tertiary care level and were able to provide guideline-based [5] patient management. The changes made during the revisions of these guidelines in 2000 and 2007, respectively, were taken into account. The number of patients enrolled by these centers (median: 28, IQR 21–65, range 3–150) varied considerably, as four centers participated in both projects, and some centers joined the second project with just few weeks remaining for patient inclusion. Hospital mortality for patients with severe TBI was significantly lower during the 2009–2010 projects (28 vs. 37 %; *P* = 0.005). However, Glasgow Coma Scale (GCS) score was significantly lower (5.4 vs. 5.9; *P* = 0.022) and Abbreviated Injury Score (AIS) for the region “head” was significantly higher (4.2 vs. 3.9; *P* < 0.001) in the patients from the 2002–2005 projects; thus, “period of enrollment” was not significantly associated with outcomes in the logistic regression analysis.

### Treatment process

Treatment in the field was provided by emergency physicians. All patients had quick examination with documentation of vital signs. Rapid sequence intubation facilitated by hypnotics and relaxants, ventilation, treatment of hemorrhage, and fluid resuscitation was done as appropriate. After hospital admission each patient was examined by a trauma team (anesthesiologists, trauma surgeons, and/or neurosurgeons, radiologists, nurses), and a computed tomography (CT) scan was done. The patients then underwent surgery as appropriate and/or were admitted to the intensive care unit (ICU). Intensive care was provided by anesthesiologists in cooperation with neuro or trauma surgeons.

### Data collection

Basic demographic data of the patient, cause and location of trauma, prehospital status and treatment, mechanism and severity of trauma [AIS, Injury Severity Score (ISS)], results of CT scans, results of lab testing, and data on surgical procedures and outcomes was recorded prospectively. Prehospital data were documented by paramedics and then transferred into the databases. Summarized CT findings [i.e., data on basal cisterns (open/compressed/absent), midline shift, main findings (edema, hematoma, contusions, etc.), Marshall classification] were entered into a separate CT page in the databases. No central review of CT scans was done in the first project. Central review of the CT scans was done in the second project; a radiologist and a trauma surgeon checked the accuracy of the data entered into the database. Data on duration of various treatments, on complications, and on outcomes were collected at discharge from the ICU, at hospital discharge, and at 6 months after injury. The Glasgow Outcome Scale (GOS) score at 6 months after injury was evaluated by phone calls to the patients or their relatives. Data were collected by local research fellows. Data quality was monitored by INRO project managers. They reported data problems to the local researchers who then submitted the missing or corrected values. Personal data protection was observed and the identifiers were kept separately from the data.

### Data analysis

All patients who had an AIS “head” (AIS_H_) < 6 and survived at least until admission to the ICU were included. Data on trauma mechanism, trauma severity, CT findings, treatment, and outcomes were retrieved for each patient. The 6-point Rotterdam CT score [6] was used to classify CT findings and to calculate probability of mortality according to this score. The prognostic scores developed by Hukkelhoven et al. [7] were used to estimate probability of hospital death (*P*_D_) and probability of unfavorable long-term outcome (*P*_U_). To describe long-term outcomes the GOS [8] was used. “Favorable outcome” was defined as a GOS score of five or four; “unfavorable outcome” was defined as a GOS score of three or less at 6 months after trauma. Patients were assigned to the group “TBI + injury” if they had one or more extracranial injuries with an AIS > 2. Patients were assigned to the group “TBI isolated” if they had no extracranial injuries with an AIS > 2. The differences between these two groups of patients were analyzed.

### Statistical analysis

Analyses were done using the software provided by P. Wessa (Free Statistics Software version 1.1.23-r6, http://www.wessa.net). Two-tailed *t* test, Fisher’s exact test, and χ^2^-test were done as appropriate to identify differences between the groups. To check for associations with outcomes we constructed logistic regression models for hospital death and favorable long-term outcome where outcomes were corrected for confounders, with backward exclusion of non-significant (*P* > 0.1) parameters. Age, gender, trauma mechanism, number of injured body regions with AIS > 2, ISS, AIS_H_, GCS score, pupillary reactivity, presence of hypoxia and hypotension, Rotterdam CT score, and requirement of neurosurgery or extracranial surgery were considered possible confounders. The models were calculated for both groups individually, and for the whole sample as well. Data are presented as means with standard deviations, or as proportions. A *P* value of <0.05 was considered statistically significant.

## Results

There were 863 data sets in the database. Of these, hospital outcomes were missing in 17 (1.9 %) patients, 10 (1.2 %) patients died prior to ICU admission, data on additional injuries were missing in 14 (1.6 %) patients, and 55 (6.4 %) had an AIS_H_ of six. This left 767 patients for analysis; of these, 403 (52.5 %) had isolated TBI, and 364 (47.5 %) had concomitant injuries. There was no significant “center effect”; all centers that enrolled >15 patients had mortality rates within the expected ranges. Values were outside these ranges in two centers that enrolled fewer patients, but this could be an effect of the low number of patients.

### Demographic data (Table 1)

Hospital mortality was 2.8 % lower in patients with concomitant injuries (n.s.). In both groups, most patients were male. Mean age was significantly higher in patients with isolated TBI (*P* < 0.001). It was also higher in females from both groups (n.s.). A significant (*P* < 0.01) increase in hospital mortality was seen with increasing age, but there was no difference in mortality rates between the groups. With regard to trauma mechanism falls and traffic-related accidents were most common in both groups. There were no significant differences in mortality rates for any of the mechanisms.

**Table 1:** Gender, age and trauma mechanism

	TBI + injury		TBI isolated		Total		*P* value
	*n*	% mort	*n*	% mort	*n*	% mort	
Patients	364	27.2	403	30.0	767	28.7	0.42
Female	85	27.1	117	33.3	202	30.7	–
Male	279	27.2	286	28.7	565	28.0	–
% female	23		29		26		
Age	Mean	SD	Mean	SD	Mean	SD	
Females	45.9	23.3	60.8	22.4	54.5	23.9	–
Males	43.0	20.4	49.5	21.4	46.3	21.1	–
All patients	43.6	21.1	52.8	22.2	48.5	22.2	–
Trauma mechanism	*n*	% mort	n	% mort	n	% mort	
Fall < 3 m	52	36.5	180	41.7	232	40.5	0.52
Fall > 3 m	56	32.1	23	17.4	79	27.8	0.27
Traffic-related	197	23.9	99	21.2	296	23.0	0.66
Sports-related	25	12.0	33	9.1	58	10.3	0.99
Work-related/no falls	7	28.6	11	18.2	18	22.2	0.99
Violence	3	33.3	14	35.7	17	35.3	0.99
Other	19	31.6	21	23.8	40	27.5	0.73
Unknown	5	60.0	22	27.3	27	33.3	0.30
Total	364	27.2	403	30.0	767	28.7	0.42
Type of trauma							
Blunt	327	28.1	375	29.9	702	29.1	0.62
Penetrating	27	18.5	18	44.4	45	28.9	0.09
Unknown	10	20.0	10	10.0	20	15.0	0.99
Total	364	27.2	403	30.0	767	28.7	0.42

The *P* value relates to the mortality difference between patients with isolated TBI and patients with concomitant injuries

*TBI* traumatic brain injury

### Trauma and TBI severity (Table 2)

The ISS was significantly higher in patients with concomitant injuries (*P* < 0.001). There was an increase in the severity of concomitant injuries for increasing values of AIS_H_: the values for ISS (calculated without AIS_H_) were 19.7 ± 8.6 (AIS_H_ = 2), 22.8 ± 11.0 (AIS_H_ = 3), 20.0 ± 10.3 (AIS_H_ = 4), and 27.9 ± 16.6 (AIS_H_ = 5), respectively (*P* = 0.1, n.s.). Mean AIS_H_ and mean GCS scores were not different. Within each group ISS and AIS_H_ were significantly higher and GCS scores were significantly lower in non-survivors. In both groups, most of the patients had severe TBI: only 17/767 [2.2 %; if (AIS_H_ > 2) is used as definition] or 95/767 [12.4 %; if (GCS score >8) is used as definition] of the patients had moderate TBI. In patients with AIS_H_ = 2 mortality was significantly lower in those with isolated TBI. Increasing GCS scores were associated with significant decreases in mortality rates in both groups. In both groups, patients with reactive pupils had significantly lower mortality rates but there were no significant differences in mortality rates between the groups. The same was true for absence of prehospital hypotension and hypoxia, respectively. Incidences of aspiration, use of anticoagulants, and comorbidities were not significantly different between the groups, and these had no significant effects upon mortality. The predicted *P*_D_ was 29.2 ± 20.5 % for patients with concomitant injuries, and was 33.4 ± 21.5 % for patients with isolated TBI (*P* = 0.06), and the *P*_U_ values were 50.9 ± 24.0 % and 54.9 ± 24.6 %, respectively (*P* = 0.02).

**Table 2:** Trauma severity

	TBI + injury		TBI isolated		Total		*P* value
	Mean	SD	Mean	SD	Mean	SD	
ISS	34.4	11.0	18.2	5.5	25.9	11.8	–
AIS_H_	3.95	0.66	4.06	0.68	4.01	0.67	–
GCS score	5.62	2.68	5.57	2.77	5.59	2.72	–
AIS_H_	*n*	% mort	*n*	% mort	*n*	% mort	
2	9	33.3	8	12.5	17	23.5	0.05
3	62	14.5	58	6.9	120	10.8	0.24
4	231	19.9	240	24.2	471	22.1	0.27
5	62	66.1	97	59.8	159	62.3	0.50
Total	364	27.2	403	30.0	767	28.7	0.42
GCS score							
3	132	41.7	148	43.9	280	42.9	0.72
4	24	29.2	39	61.5	63	49.2	0.02
5	29	27.6	34	23.5	63	25.4	0.7
6	57	29.8	53	20.8	110	25.5	0.38
7	41	14.6	41	12.2	82	13.4	0.99
8	38	5.3	36	19.4	74	12.2	0.08
9–12	43	9.3	52	1.9	95	5.3	0.17
Total	364	27.2	403	30.0	767	28.7	0.42
Marshall score							
Diffuse injury 1	43	11.6	19	5.3	62	9.7	0.44
Diffuse injury 2	97	24.7	73	13.7	170	20.0	0.08
Diffuse injury 3	39	35.9	26	38.5	65	36.9	0.83
Diffuse injury 4	5	60.0	3	66.7	8	62.5	–
Evacuated lesion	90	32.2	173	35.3	263	34.2	0.62
Non-evacuated lesion	87	25.3	107	32.7	194	29.4	0.26
Not determined	3	66.7	2	100.0	5	80.0	–
Total	364	27.2	403	30.0	767	28.7	0.42

The *P* value relates to the mortality difference between patients with isolated TBI and patients with concomitant injuries

*TBI* traumatic brain injury; *AIS*_*H*_ Abbreviated Injury Score for the region “head”; *ISS* Injury Severity Score; *GCS* Glasgow Coma Scale

### CT findings

Mortality increased significantly with increasing Rotterdam CT scores (*P* < 0.001), but there were no significant mortality differences between the groups. The mortality rates were lower than those predicted by the Rotterdam score; a significant correlation (*y* = 15.963*x* − 2.388; *R* ^2^ = 0.992; *P* = 0.03) between observed and predicted values was found for patients with isolated TBI only. With regard to predominant lesions, subdural hematoma was observed most frequently, followed by contusion, EDH and subarachnoid hemorrhage. The overall distribution of predominant lesions was not significantly different between the groups. There were no differences in mortality between the two groups within the different classes of the Marshall CT score (Table 2).

### Treatment factors (Table 3)

Most patients were admitted directly to the study centers; mortality was lower in the 125 patients (16.3 %) with indirect transfer (*P* = 0.002). Air and ground transport were associated with comparable mortality rates. Patients who required prehospital airway management had a significantly (*P* = 0.008) higher mortality. There were no differences regarding the intervals between admission and start of CT scan, and between start of CT scan and start of surgery. The majority of the patients (*n* = 504; 65.7 %) were managed conservatively and had either no surgical procedure or insertion of an ICP monitoring device only. Mortality was lower in the patients who had primary craniectomy than in those who had craniotomy (*P* = 0.053). The requirement for extracranial surgery was associated with significantly higher mortality in patients with concomitant injuries. Duration of ventilation (12.8 ± 11.2 vs. 10.1 ± 10.8 days), ICU stay (22.0 ± 18.5 vs. 17.4 ± 16.3 days), and hospital stay (42.0 ± 39.4 vs. 28.2 ± 25.6 days) were significantly shorter in survivors with isolated TBI than in those with concomitant injuries. No significant differences regarding these parameters were found in non-survivors.

**Table 3:** Treatment

	TBI + injury		TBI isolated		Total		*P* value
	*n*	% mort	*n*	% mort	*n*	% mort	
Indirect transfer							
No	321	29.3	321	32.4	642	30.8	0.44
Yes	43	11.6	82	20.7	125	17.6	0.23
Mode of transport							
Air	175	26.3	139	26.6	314	26.4	0.99
Ground	177	29.9	248	33.5	425	32.0	0.46
Unknown	12	0.0	16	6.3	28	3.6	0.99
Prehospital intubation							
No	76	21.1	148	22.3	224	21.9	0.87
Yes	288	28.8	255	34.5	543	31.5	0.17
Neurosurgery							
No neurosurgery	107	27.1	125	24.8	232	25.9	0.76
ICP monitoring only	167	24.6	105	27.6	272	25.7	0.57
ASDH evacuation	45	33.3	121	39.7	166	38.0	0.48
EDH evacuation	28	25.0	27	29.6	55	27.3	0.77
ICH evacuation	5	40.0	9	11.1	14	21.4	0.51
>1 lesion evacuated	7	42.9	14	14.3	21	23.8	0.28
Primary decompressive surgery	5	40.0	2	100.0	7	57.1	0.43
Total	364	27.2	403	30.0	767	28.7	0.42
Secondary decompressive surgery	6	50.0	5	60.0	11	54.5	0.86
Technique							
Decompressive surgery	11	54.5	8	62.5	19	57.9	0.99
Craniectomy	36	27.8	63	28.6	99	28.3	0.99
Craniotomy	43	30.2	102	37.3	145	35.2	0.45
Total	90	30.2	173	37.3	263	28.7	0.94
ICP monitoring							
No	124	28.2	177	25.4	301	26.6	0.60
Yes	240	26.7	226	33.6	466	30.0	0.11
Extracranial surgery							
No	201	28.9	376	32.2	577	31.0	0.45
Yes	163	25.2	27	0.0	190	21.6	0.002

The *P* value relates to the mortality difference between patients with isolated TBI and patients with concomitant injuries

*TBI* traumatic brain injury; *ICP* intracranial pressure; *ASDH* acute subdural hematoma; *EDH* epidural hematoma; *ICH* intracerebral hemorrhage

### Concomitant injuries (Table 4)

Injuries to the thoracic region and to extremities were associated with higher, isolated injuries to the face with lower mortality. None of the observed mortality rates was significantly different from the average mortality for the whole group. The overall incidences of associated injuries were: 191 (24.9 %) patients had thoracic, 166 (21.6 %) had facial, 154 (20.1 %) had extremity, 58 (7.6 %) had spinal, 20 (2.6 %) had abdominal, and 6 (0.8 %) had external injuries. Of the 58 spinal injuries, 20 (34.5 %; 2.6 % of all) were cervical, 28 (48.3 %; 3.7 % of all) were thoracic, and 10 (17.2 %; 1.3 % of all) were lumbar spine injuries.

**Table 4:** Concomitant injury pattern

Outcome	Alive	Dead	Total	% of all	% mort
	*n*	*n*	*n*		
Injured body regions					
Face	55	13	68	18.7	19.1
Thorax	36	15	51	14.0	29.4
Extremities	24	16	40	11.0	40.0
Thorax + extremities	13	10	23	6.3	43.5
Face + thorax	19	2	21	5.8	9.5
Face + extremities	16	4	20	5.5	20.0
Face + thorax + extremities	12	7	19	5.2	36.8
Thorax + abdomen	14	2	16	4.4	12.5
Thorax + abdomen + extremities	8	5	13	3.6	38.5
Thoracal spine	9	2	11	3.0	18.2
Abdomen	8	2	10	2.7	20.0
Other	51	21	72	19.8	29.2
Total	265	99	364	100.0	27.2

All injuries or combinations of injuries with an incidence of >2 % in the 364 patients with traumatic brain injuries and concomitant injuries are listed

### Treatment of concomitant injuries (Table 5)

More than half of the injuries did not require surgical interventions. The number of surgical procedures required was not significantly associated with mortality rates. Orthopedic procedures involving extremities or pelvic region were done most frequently. Abdominal surgery and thoracic surgery were associated with higher mortality rates.

**Table 5:** Surgery in 364 patients with TBI plus concomitant injuries

Outcome	Alive	Dead	Total	% of all	% mort
Number of surgical procedures					
0	142	58	200	54.9	29.0
1	71	27	98	26.9	27.6
2	38	9	47	12.9	19.1
3	11	1	12	3.3	8.3
4	3	3	6	1.6	50.0
5		1	1	0.3	100.0
Total	265	99	364	100.0	27.2
Region of surgery					
Lower extremity	49	19	68	26.5	27.9
Face	43	7	50	19.5	14.0
Thorax	32	16	48	18.7	33.3
Upper extremity	29	6	35	13.6	17.1
Abdomen	18	13	31	12.1	41.9
Pelvis	5	3	8	3.1	37.5
Cervical spine	6	1	7	2.7	14.3
Thoracic spine	7	0	7	2.7	0.0
Lumbar spine	3	0	3	1.2	0.0
Total	192	65	257	100.0	25.3

*SP* surgical procedures; *% of all* percentage of surgical procedures in the 364 patients with traumatic brain injury and concomitant injuries; *% of SP* percentage of all surgical procedures

### Outcomes

The observed hospital mortality was 27.2 % for patient with concomitant injuries and 30.0 % for patients with isolated TBI, while *P*_M_ values were 29.2 ± 20.5 % and 33.4 ± 21.5 %, respectively. The observed vs. expected ratio (O/E ratio) for mortality was 0.93 for patients with concomitant injuries (=25 unexpected survivors), the O/E ratio for mortality was 0.90 for patients with isolated TBI (=40 unexpected survivors). Main causes of death in patients with concomitant injuries were brain death (51.9 %), cardiovascular problems (31.5 %), multiple organ failure (9.8 %), major hemorrhage (4.4 %), and acute respiratory distress syndrome (2.2 %). In patients with isolated TBI brain death was observed significantly more frequently (65.1 %, *P* = 0.04), and the rates of cardiovascular death (25.7 %) and multiple organ failure (5.5 %) were lower. Long-term outcome was unknown in 28 patients (14 from each group). Favorable outcome was observed in 54.4 % (198/364) of patients with concomitant injuries and in 46.2 % (186/403) of the patients with isolated TBI; this difference was significant (*P* = 0.02). Unfavorable long-term outcome was observed in 41.8 % (152/364) and 50.4 % (203/403), respectively; this difference was also significant (*P* = 0.02). The *P*_U_ predicted by the Hukkelhoven score was 50.9 ± 24 % for patients with associated injuries, and was 54.1 ± 24.1 % for patients with isolated TBI. The O/E ratio for unfavorable outcome was 0.82 for patients with concomitant injuries (=66 patients with unexpected favorable outcome), the O/E ratio for unfavorable outcome was 0.93 for patients with isolated TBI (=28 patients with unexpected favorable outcome). Factors that significantly influenced outcomes are listed in Table 6. Age, GCS score, pupillary reactivity, AIS_H_ and CT score were significant in all or almost all analyses. Isolated TBI was significantly associated with unfavorable long-term outcome. Major neurosurgery was associated with higher mortality in patients with isolated TBI, ISS was associated with worse long-term outcomes in patients with concomitant injuries.

**Table 6:** Factors that significantly (*P* < 0.01) influenced the outcomes

	Hospital death		Long-term outcome	
TBI isolated	Parameter	*P* value	Parameter	*P* value
	Age	< 0.001	Age	< 0.001
	AIS_H_	< 0.001	AIS_H_	< 0.001
	Pupils	0.002	GCS score	< 0.001
	GCS score	< 0.001	CT score	< 0.001
	CT score	0.007		
	Neurosurgery	0.003		
TBI + injury				
	Age	< 0.001	Age	< 0.001
	AIS_H_	0.004	Pupils	< 0.001
	Pupils	< 0.001	GCS score	< 0.001
	GCS score	0.005	ISS	< 0.001
All patients				
	Age	< 0.001	Age	< 0.001
	AIS_H_	< 0.001	Pupils	0.002
	Pupils	< 0.001	GCS score	< 0.001
	GCS score	< 0.001	CT score	0.001
	CT score	< 0.001	Isolated TBI	0.007

*AIS*_*H*_ Abbreviated Injury Score for the region “head”; *GCS* Glasgow Coma Scale; *CT* computed tomography; *ISS* injury severity score; *TBI* traumatic brain injury

## Discussion

The overall rates of hospital mortality and unfavorable outcomes seen in this study are comparable to the outcomes reported for European centers [9]. With regard to factors influencing outcomes, age is one of the most important. This has been demonstrated in the large study done by Hukkelhoven et al. [10], and by a number of other studies. The significant effect of age has been confirmed by our results. In addition, the effects of GCS scores on outcomes after TBI have been proven beyond doubt [11]. This association was also found in our study. In the same analysis, one or both unreactive pupils were significantly associated with poor outcome [11]. This association was also confirmed in our multivariate analysis.

In this study, monitoring of ICP was done in only 70 % of the patients with severe TBI. In some patients, this was probably due to poor prognosis. A previous study involving 82 Austrian ICUs found that ICP monitoring was not done in patients whose prognosis was either poor or good; the highest rates of ICP monitoring were found in the patients with an “intermediate” prognosis [12]. A recent study from the Netherlands reported that ICP was monitored in only 46 % of patients with severe TBI; higher age was one of the reasons not to monitor ICP [13].

One of the earlier studies on TBI and concomitant injuries [14] found that only 42 % of the patients had isolated TBI, and that concomitant injuries had effects on long-term outcomes only if they involved at least two or more body regions. A Swiss study [3] reported that 59 % of their 139 patients with EDH had isolated TBI, and that concomitant injuries had no effects on outcomes, and a study from the Germany [15] came to the same conclusion. In their analysis of a large German database, Lefering et al. [2] found significantly increased mortality rates in patients whose torso or extremity injuries had an AIS of five or six. No such effect was observed in our study, but this could be due to the much smaller sample of patients. Thus, most studies concluded that concomitant injuries had either no effect on outcomes, or had effects only if the injuries were of high severity. This is in accordance with our results; isolated TBI was actually associated with worse outcomes. A similar result was found in the study by Martins et al. [16]; in their study, mortality was significantly higher in patients with isolated TBI (37.6 vs. 27.6 %; *P* = 0.004).

In our study, mortality was significantly higher in the patients with concomitant injury and an AIS_H_ of two. This could be an effect of the fact that only patients who were admitted to the ICU were included. Thus, ICU admission of patients with an AIS_H_ of two may have been due to extracranial rather than intracranial injuries. However, it seems obvious that the effect of extracranial injuries would be more pronounced in patients with low severity of TBI. In a study on mild TBI [17] significant effects of extracranial injuries on duration and outcomes of rehabilitation were found.

Our study found a high incidence (21.6 %) of facial trauma. It has been suggested that facial fractures protect the brain from severe injury, but this has been proven wrong [18]: of the 3,040 patients with TBI, 848 (27.9 %) were found to have facial fractures, and TBI severity was not different between the patients with and without facial trauma. The rates of additionally injured regions were comparable to those published by Martins et al. [16]. Compared to our data, Rickels et al. [19] found higher rates of facial trauma (58 %) and lower rates for all other concomitant injuries; however, this study included mostly patients with mild TBI.

Regarding causes of death Kemp et al. [1] compared data from 54 non-survivors with isolated TBI to those from 81 non-survivors with concomitant injuries. Their data are not fully comparable to those from our study because they also included patients with non-survivable TBI; they found, however, that brain death occurred more frequently in cases of isolated TBI, and that respiratory failure occurred significantly more frequently in patients with multiple trauma (43.2 vs. 20.4 %). A comparable pattern was seen in our study.

Contrary to previous reports, we found that indirect transfer was associated with lower mortality rates. Hartl et al. [20] reported that indirect transfer was associated with a 50 % increase in mortality for patients with severe TBI. The difference may be due to the low number of patients with indirect transfer in our study.

### Limitations of the study

The scores used to estimate *P*_M_ and *P*_U_ have not been validated for our study population. These scores have been created from the international and North American data from the tirilazad trial [21, 22], and have been validated against the core data set of the European Brain Injury Consortium (EBIC) survey [23] and data from the Traumatic Coma Data Bank [24]. It is quite likely that our patients are comparable to those from the EBIC centers and the international arm of the tirilazad trial. There could, however, be subtle differences, and the O/E ratios estimated for our groups of patients may be incorrect.

## Conclusions

The study showed that concomitant injuries were found in nearly half of the patients. Hospital mortality was 2.8 % higher, and the rate of unfavorable outcome was 8.6 % higher in patients with isolated TBI. Concomitant injuries were associated with significantly higher mortality in the few patients with AIS_H_ = 2. Concomitant injuries were also associated with longer duration of ventilation, and longer ICU and hospital stay. We were unable to find any significant effects of treatment. The worst outcomes of patients with isolated TBI were mainly due to a significantly higher mean age. The main factors that influenced the outcomes were age, GCS score, pupillary reactivity, AIS_H_ and CT score.

## Appendix: Austrian Severe TBI Study Group

Investigators from the participating centers: H. Artmann MD (Schwarzach), N. Bauer MD (Linz UKH), F. Botha MD (Linz WJ), F. Chmeliczek MD (Salzburg LKA), G. Clarici MD (Graz Uni), D. Csomor MD (Wr. Neustadt), R. Folie MD (Feldkirch), R. Germann MD, PhD (Feldkirch), F. Gruber MD (Linz AKH), H-D. Gulle MD (Klagenfurt), T. Haidacher MD (Graz UKH), G. Herzer MD (Wr. Neustadt), P. Hohenauer MD (Salzburg LKA), A. Hüblauer MD (Horn), J. Lanner MD (Salzburg UKH), V. Lorenz MD (Wien UKH XII), C. Mirth MD (St. Pölten), W. Mitterndorfer MD (Linz AKH), W. Moser MD (Klagenfurt), H. Schmied MD (Amstetten), K-H Stadlbauer MD, PhD (Innsbruck), H. Steltzer MD, PhD (Wien UKH XII), Ernst Trampitsch MD (Klagenfurt), A. Waltensdorfer MD (Graz Uni), A. Zechner MD (Klagenfurt); INRO researchers: M. Rusnak MD, PhD (Epidemiology, Public Health), I. Janciak PhD (IT support, database management)
